# Bacterial subversion of NLR-mediated immune responses

**DOI:** 10.3389/fimmu.2022.930882

**Published:** 2022-07-28

**Authors:** Ioannis Kienes, Ella L. Johnston, Natalie J. Bitto, Maria Kaparakis-Liaskos, Thomas A. Kufer

**Affiliations:** ^1^ Department of Immunology, University of Hohenheim, Stuttgart, Germany; ^2^ Department of Microbiology, Anatomy, Physiology and Pharmacology, La Trobe University, Melbourne, VIC, Australia; ^3^ Research Centre for Extracellular Vesicles, La Trobe University, Melbourne, VIC, Australia

**Keywords:** PAMP, DAMP, infection, tolerance, pathogens, NLRs, inflammation, inflammasome

## Abstract

Members of the mammalian Nod-like receptor (NLR) protein family are important intracellular sensors for bacteria. Bacteria have evolved under the pressure of detection by host immune sensing systems, leading to adaptive subversion strategies to dampen immune responses for their benefits. These include modification of microbe-associated molecular patterns (MAMPs), interception of innate immune pathways by secreted effector proteins and sophisticated instruction of anti-inflammatory adaptive immune responses. Here, we summarise our current understanding of subversion strategies used by bacterial pathogens to manipulate NLR-mediated responses, focusing on the well-studied members NOD1/2, and the inflammasome forming NLRs NLRC4, and NLRP3. We discuss how bacterial pathogens and their products activate these NLRs to promote inflammation and disease and the range of mechanisms used by bacterial pathogens to evade detection by NLRs and to block or dampen NLR activation to ultimately interfere with the generation of host immunity. Moreover, we discuss how bacteria utilise NLRs to facilitate immunotolerance and persistence in the host and outline how various mechanisms used to attenuate innate immune responses towards bacterial pathogens can also aid the host by reducing immunopathologies. Finally, we describe the therapeutic potential of harnessing immune subversion strategies used by bacteria to treat chronic inflammatory conditions.

## 1 Introduction

Bacteria have evolved complex interactions with mammals, resulting in both beneficial and detrimental effects for the host. On the host side, molecular sensing systems of the innate immune system detect non-host components and products, typically conserved structural components of microbes, such as peptidoglycan (PGN), lipopolysaccharides, and lipoteichoic acids. These microbe-associated molecular patterns (MAMPs) activate receptors on and within host cells, referred to as pattern-recognition receptors (PRRs), to trigger signal transduction events ultimately leading to the production of immune mediators and anti-microbial peptides (reviewed in (1)).

While overwhelming colonisation of the host with bacteria must be avoided and most organs are regarded as sterile, the host also depends on bacteria, their MAMPs and metabolites for proper function of its immune system and the development and homeostasis of its protective barrier surfaces. This is probably best exemplified by the well-studied intestinal barrier, where a wealth of recent studies show that the gut microbiota provides essential signals that also affect the regulation of systemic immune responses (2). Besides providing such beneficial effects, overwhelming replication of bacteria in the host would impair its survival. Moreover, some pathogenic bacteria can actively invade the host *via* the expression and use of virulence factors that enable them to overcome physical and immunological barriers (3). In addition, some pathogenic bacteria can also promote their uptake by host cells and live and replicate in cellular compartments such as endosomes, or in some cases replicate and move freely in the cytosol of the host cell. As such, these organisms present a threat to the host and their replication needs to be timely and tightly controlled by the host’s immune response.

On the other side, pathogens try to subvert immune responses for their replicative benefit. This system is highly dynamic and driven by the rapid evolution of pathogens and the adaptation of the host. This can be illustrated by the paradigm of the “Red-Queen” from Lewis Carrol’s fairy tale that is often used to describe the arms race between pathogens and their hosts (4). However, such an immuno-centric view lacks consideration of the fact that an uncontrolled, and overwhelming immune response focused on completely eradicating pathogens could come at the cost of significant collateral damage of host tissue, eventually leading to severe pathologies. Thus, this arms-race between host and pathogens needs to be controlled and tightly regulated.

Indeed, the host and its surrounding microbes have evolved for fine-tuning of the immune response, in order to guarantee sufficient restriction of the invading pathogen and assure integrity and functionality of the host, while at the same time limiting harmful tissue damage and immunopathology. This is described by the concept of resistance and tolerance, where resistance refers to the capacity of elimination of the pathogen by the immune response, and tolerance to a state of acceptance of some colonisation and increased tissue homeostasis to avoid immunopathology (5).

Historically, the field of infection biology research has focussed on examining the beneficial roles of the immune system to defend against microbes and to understand how pathogens can use subversion strategies to overcome host immune responses. However, within the past decade, our understanding of immunomodulation of innate immune responses and its importance in promoting tolerance to infection and host fitness is emerging. Recent data suggests that during the evolution of humans, attenuation of cytokine responses towards intracellular pathogens might have been a key event to guarantee survival and fitness of the host (6).

Charles Janeway’s idea that the host detects pathogens using germline encoded receptors of the innate immune system to trigger inflammation and to introduce adaptive immunity (7) paved the way for the identification of a wealth of PRRs and deciphering their cellular signalling pathways (8). In humans we have several classes of PRRs that represent both membrane anchored receptors, such as the Toll-like receptors (TLRs) (9) or C-type lectin receptors (CLRs) (10) and intracellular receptors such as the NOD-like receptors (NLRs) (11), RIG-I like receptors (RLRs), and cyclic GMP-AMP synthase (cGAS) (12). All these PRRs have different specificities that collectively cover the detection of a broad range of MAMPs derived from bacterial, fungal and metazoan pathogens. Activation of PRRs leads to the induction of cellular signalling events that ultimately triggers the release of anti-microbial substances such as antibacterial peptides, the production of cytokines, recruitment of immune cells and the induction of adaptive immune responses.

Amongst the PRR families, Nod-like receptor (NLR) proteins gained interest due to the fact that this family of 22 proteins in humans serve diverse functions in innate immunity (13). NLRs show a typical tripartite structure hallmarked by a central oligomerization domain with nucleotide binding capacity, a C-terminal leucine rich repeat (LRRs) domain that is also found in other PRRs such as TLRs, and different N-terminal domains that define their signalling function. NOD1 and NOD2 were the first NLRs to be described as PRRs and to serve as intercellular receptors for invasive bacteria (14). They induce transcriptional reprogramming by their CARD domains that interact with the Receptor Interacting Serine/Threonine Kinase 2 (RIP2) to induce Mitogen-activated protein kinase (MAPK) and I kappa-B Kinase (IKK) activation (14). In contrast, many PYD domain containing NLRs form inflammasomes that act as a scaffold for the activation of caspase-1, which subsequently can process pro-IL-1β, pro-IL-18 and gasdermin D to induce release of the potent pro-inflammatory mediators IL-1β and IL-18 (15). Of note, inflammasomes not only respond to MAMPs but are also activated by perturbance of cellular membrane integrity and danger-associated molecular patterns (DAMPs) which are factors that are released upon tissue and cell disintegration. The innate immune system thus can detect pathogen-induced damage of tissues and cells, and also the perturbation of cellular pathways (16, 17). This indirect recognition of pathogens as a result of changes in cellular signalling and induction of cellular stress is also referred to as effector-triggered-immunity (ETI) in relation to the immune response triggered by pathogen effector proteins in plants (18, 19).

Here we focus on well-studied members of the NLR-family, a class of host PRRs that are expressed in the cytosol. We will discuss our current understanding of their roles as PRRs for bacteria, but also take a closer look at the mechanisms used by bacterial pathogens to overcome NLR-mediated responses. In view of the need of a well-adapted immune response towards pathogens to avoid immunopathologies, we hypothesise that such adaptations of bacteria did not evolve solely to assure better colonisation and survival in the host, but also to support fitness of the host for the benefit of the bacteria.

## 2 Non inflammasome NLRs

### 2.1 NOD1 and NOD2 detect bacterial peptidoglycan resulting in a proinflammatory immune response

**Figure 1:**
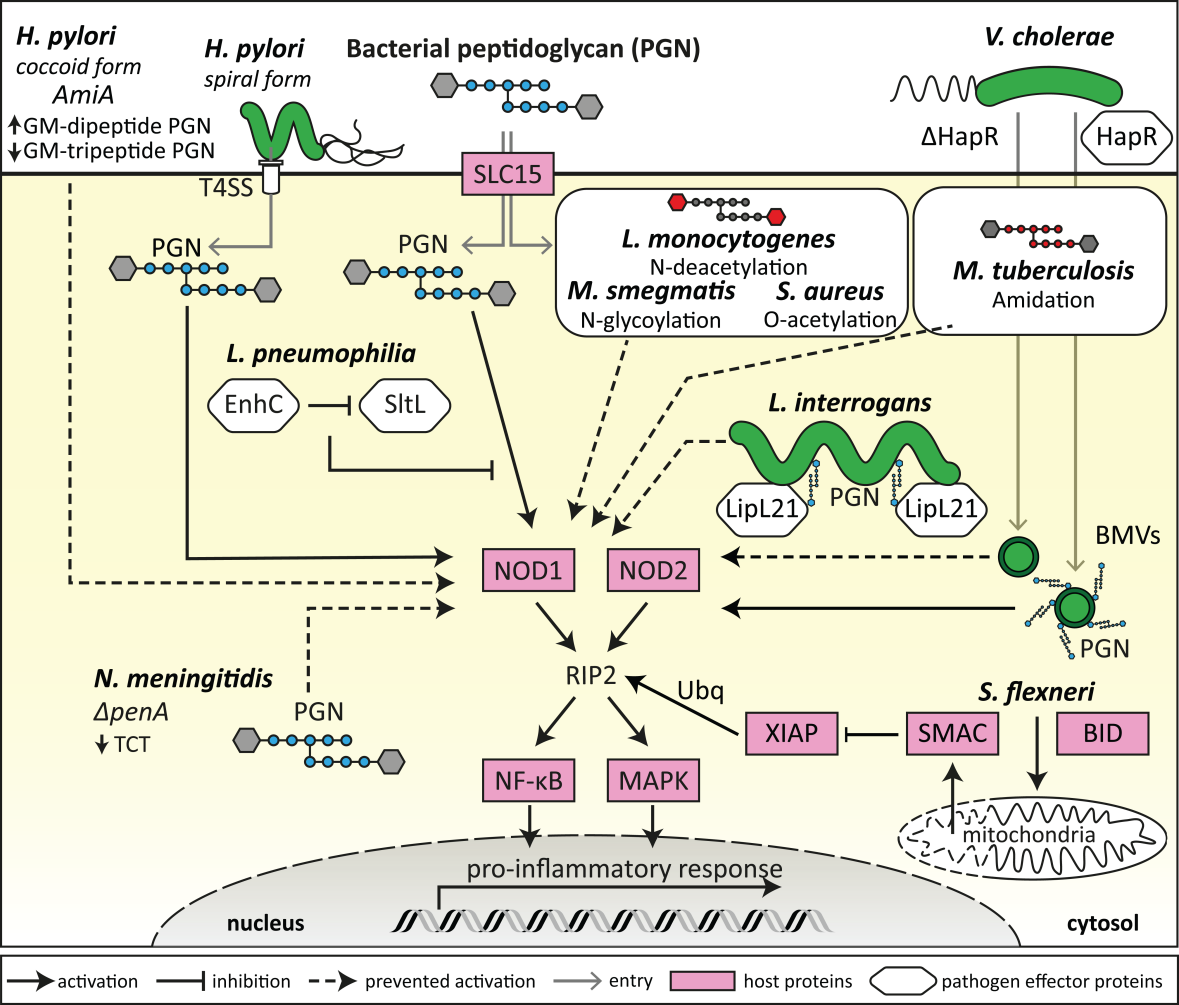
Bacterial evasion of NOD1 and NOD2 detection. Bacteria can modify their morphology and metabolism to evade detection by NODs by a range of mechanisms. This includes *H. pylori* transitioning from spiral to coccoid morphology, which results in decreased GM-tripeptide accumulation, and deletion of *penA* by *N. meningitidis* which results in decreased TCT tetrapeptide peptidoglycan (PGN) moieties, ultimately reducing the availability of NOD ligands to prevent NOD1 activation. Some bacteria express proteins that can block the enzymatic release of specific NOD-activating PGN moieties (*L. pneumophila*) or can sequester NOD ligands to the bacterial surface (*L. interrogans*), thus preventing NOD1 and NOD2 activation. Several bacterial strains, such as *S. aureus, M. tuberculosis, M. smegmatis* and *L. monocytogenes*, have processes to modify their PGN in order to evade NOD1/2 detection and activation, resulting in an attenuated proinflammatory response. Bacteria also release bacterial membrane vesicles (BMVs) containing PGN that can activate NODs, and bacterial expression of proteins such as HapR (*V. cholerae*) can alter the PGN content of BMVs and therefore modulate NOD1 and NOD2 activation. Bacteria such as *S. flexneri* can induce BID-dependent selective permeability of the mitochondria, resulting in the release of SMAC, which blocks XIAP ubiquitination of RIP2 downstream of NOD1 and NOD2 activation.

Among the non-inflammasome forming NLRs that regulate inflammation, NOD1 and NOD2 are the most well characterised receptors. NOD1 and NOD2 detect bacterial PGN, specifically the synthetic minimal PGN moieties γ-D-Glu-*meso* diaminopimelic acid (iE-DAP) and muramyl dipeptide (MDP) respectively (20, 21). Although NOD1 and NOD2 are closely related receptors that both detect specific components of bacterial PGN, NOD1 is typically expressed broadly throughout tissues at varying levels, however NOD2 expression is mostly restricted to monocytes (22–24). NOD1 and NOD2 are expressed by non-vertebrate and vertebrate species, and several amino acids are conserved in NOD1 and NOD2 which are especially notable in the LRR domains, which may be indicative of evolutionarily conserved ligand binding or recognition regions (25). Murine and human NOD1 differ in their ability to detect some PGN moieties, whereby human NOD1 requires a tripeptide for activation, and murine NOD1 requires a tetrapeptide (26). Interestingly, some bacteria such as commensal *Enterococcus* species have been shown to modify their release of PGN fragments which resulted in increased activation of murine NOD2 (27). Delivery of NOD1 and NOD2 PGN ligands into the host cell cytosol is required for their activation. As such, PGN ligands have been shown to enter host cells using a variety of mechanisms, either *via* endosomal peptide transporters of the SLC15 family (28, 29), by injection of PGN by bacterial type 4 secretion systems (T4SS) (20, 30), and by the entry of bacterial membrane vesicles (BMVs) into host cells (**Figure 1**) (31, 32). After PGN detection, NOD1 and NOD2 have been shown to associate with endosomal membranes (33, 34), which are hypothesised to be the site for NOD complex formation, coined the “nodosome” (35, 36). Before activation, NOD1 and NOD2 are thought to exist as monomers in an autoinhibited state when inactive in the cytosol, however upon ligand recognition, NOD1 and NOD2 self-oligomerise *via* their central NACHT domain (23, 37). Once activated, NOD1 and NOD2 recruit the kinase RIP2, that acts as a scaffolding protein for downstream signalling mediators and the formation of the nodosome (37). This results in downstream activation of NF-κB and MAPK signalling pathways, which ultimately leads to the production of inflammatory cytokines and chemokines (**Figure 1**) (23, 37–39). RIP2-mediated signalling is dependent on the recruitment of inhibitor of apoptosis protein (IAP) E3-ligase family members including X-linked IAP (XIAP), cellular IAP-1 (cIAP1) and cIAP2, and tumour necrosis factor (TNF) receptor associated factors such as TRAF2, TRAF5 and TRAF6 (40, 41).

NOD1 and NOD2 specifically require the action of the ubiquitin ligase XIAP for RIP2-induced activation of downstream kinases, which was confirmed in several independent studies (40, 42–44). XIAP itself is inhibited by the mitochondrial effector SMAC to control apoptosis and inflammation (45, 46). However, the enteroinvasive pathogen *Shigella flexneri*, for which NOD1 is a critical sensor, uses a sophisticated system to target XIAP by inducing a selective permeability of the mitochondria that leads to the release of SMAC but not of the apoptosis inducing cytochrome c in a BID-dependent manner (**Figure 1**) (47). It remains to be seen if this strategy to dampen NOD1 signalling is also used by other pathogens. Of note, targeting of the RIP2-XIAP interaction to block NOD1/2 induced inflammatory signals is emerging as a therapeutical option, as small compound XIAP- and RIP2-inhibitors limit inflammation by blocking XIAP-RIP2 interactions (48). Such drugs could be useful to dampen excessive or chronic inflammation resulting from inflammatory and infectious diseases. Overall, the most efficient strategy to subvert NOD1/2 detection is the targeting of signalling downstream of NOD1/2. Inhibition of NF-κB and MAPK signalling for example are a common theme of many bacterial pathogens that evolved secreted effectors to target these pathways. In this review, we will focus our discussion on bacterial mechanisms of NOD1/2 specific subversion. For further details summarising the general inhibition of inflammatory pathways by bacteria, we refer the reader to the following detailed reviews (49, 50).

### 2.2 Stress sensing, disruption of the actin cytoskeleton and S1P sensing affect NOD1 and NOD2 signalling

In addition to the detection of bacterial PGN by NODs, NOD1 and NOD2 have also been shown to be important for the clearance of bacteria by autophagy in several studies (51, 52). Furthermore, NOD1 and NOD2 activation is also linked to endoplasmic reticulum (ER) stress and inflammatory diseases, and therefore NOD1 and NOD2 are thought to have complex roles in inflammatory signalling (38, 53–58). Specifically, bacterial induction of ER stress and cytoskeletal perturbations are linked to modulation of NOD1 and NOD2 signalling and are also the target of bacterial subversion mechanisms of NOD1/2 activation. NOD2 was initially discovered due to its involvement in Crohn’s disease (CD) through genetic linkage studies (59), with a loss-of-function mutation being the most common mutation associated with CD (60). More recently, further evidence has demonstrated that NOD1 and NOD2 are also linked to several inflammatory diseases in addition to CD, including Type 2 Diabetes (T2D), and asthma (20, 21, 38, 53–55). ER stress has more recently been identified as a major contributor to the pathology of inflammatory diseases including CD and T2D (56, 61–63), with NOD1 and NOD2 activation also being shown to be linked to ER stress (56). Thus, NOD1 and NOD2 not only act as sensors for PGN but are also activated indirectly by cellular stress responses that can be induced by pathogens. Cells respond to cellular stress with a complex program that involves the generation of the lipid sphingosine-1-phosphate (S1P) (57). S1P is a bioactive metabolite that has been shown to target TRAF2 and cIAP (64) but can also interact directly with NOD1 and NOD2 to induce IL-6 and IL-8 expression in a NOD1/2-dependent manner (57). Additionally, ER stress induced by thapsigargin and dithiothreitol were found to trigger the production of IL-6 in a NOD1/2 dependent manner (56). This suggests that S1P might be a common factor that links cellular stress to NOD1/2-induced inflammation. Furthermore, different signalling components downstream of the unfolded protein response (UPR) during ER stress might also contribute to NOD1 activation. For example, treatment of HeLa cells that expressed an NF-κB reporter with tunicamycin, a chemical that interferes with *N*-linked glycosylation to induce ER stress, did not affect the ability of NOD1 to induce NF-κB activation (58). However, treatment of HeLa cells with thapsigargin, which depletes ER calcium stores to induce ER stress, resulted in NOD1 activation when cells were stimulated with the NOD1 agonist C12-iE-DAP in combination with the *Salmonella enterica* serovar Typhimurium effector protein SopE (58). It should be noted that this sensing mechanism can also lead to adverse effects, as it was shown that ER stress can increase the susceptibility of HeLa cells to infection with *S*. Typhimurium, likely due to NOD1 hyperresponsiveness (58). Therefore, the indirect activation of NOD1 and NOD2 by cellular stress signalling may be another potential target for bacterial subversion mechanisms.

In addition to the direct activation of NOD1 and NOD2 during stress signalling, NOD1 and NOD2 have also been shown to be activated as a result of actin cytoskeleton perturbations (44, 65, 66). For example, it was demonstrated that NOD1 is recruited to the cell membrane at the site of bacterial entry, and that NOD1 and NOD2 recruit the autophagy protein ATG16L1 to direct autophagy of invading bacteria (52, 66). NOD1 also interacts with the cofilin phosphatase SSH1, that regulates the actin severing activity of cofilin, which contributes to NOD1 activation upon infection with *S. flexneri* (44). NOD1 was also found to be linked to activation of the small GTPases Rac1 and CDC42 by bacterial virulence factors, such as SopE from the enteric pathogen *S*.* *Typhimurium (67). In monocytes treated with MDP, NOD2 was shown to be recruited to the plasma membrane by a mechanism which required the RhoGTPase Rac1 and Rho guanine nucleotide exchange factor 7 (Rho GEF7) (68). Additionally, NOD2 was also reported to interact with a cytoskeletal protein, vimentin, to regulate NF-κB activation and autophagy (69). In this study, it was demonstrated that some NOD2 variants with mutations in the LRR domain, responsible for detection of PGN (20), were unable to bind vimentin which correlated with the inability of NOD2 to localise to the plasma membrane and initiate the cellular degradation pathway of autophagy (69). Furthermore, a recent study demonstrated that NOD2-MDP binding is enhanced the action of the small GTPase ADP-ribosylation factor 6 (Arf6) which contributes to membrane anchoring during activation of NOD2 (70). These indirect pathogen sensing mechanisms of NOD1/2, by monitoring actin and small GTPase activity in host cells, might also be subject for bacterial subversion and adaption to the host. *Klebsiella pneumoniae*, for example, has been found to dampen the inflammatory immune response in an indirect NOD1-dependent manner, by inhibiting Rac1 activation. This triggers NOD1-mediated upregulation of CYLD and mitogen-activated protein kinase 1 (MKP-1) expression, in turn attenuating IL-1β induced IL-8 production (71). In this way, *K. pneumoniae* utilises NOD1 to reduce the production of proinflammatory cytokines and chemokines to prevent bacterial clearance (71). Bacteria may also use several direct mechanisms to modulate NOD1 and NOD2 signalling, such as the release of PGN-containing bacterial membrane vesicles (BMVs).

### 2.3 Bacterial membrane vesicles affect NOD1 and NOD2 signalling

BMVs have been shown to package PGN cargo and can enter host cells to modulate NOD1 and NOD2 signalling (31–33, 72–75). Specifically, deletion of the quorum sensing regulator HapR, involved in *Vibrio cholerae* virulence, can reduce the packaging of PGN cargo within BMVs (72). Furthermore, stimulation of host cells using BMVs produced by HapR deletion mutants resulted in attenuated NOD1 and NOD2 responses compared to stimulation with wild-type *V. cholerae* BMVs, further pinpointing the effects of PGN packaging within BMVs and their ability to activate NOD1 and NOD2 (**Figure 1**) (72). Interestingly, HapR deletion did not affect the bacterial membrane of *V. cholerae*, despite the influence of HapR deletion on the PGN content of BMVs, which may indicate selective PGN packaging within *V. cholerae* BMVs as a mechanism to modulate NOD1/2 activation (72). *Porphyromonas gingivalis*, a periodontal pathogen, was also shown to produce BMVs that induce NOD1 and NOD2 activation (75). However, BMVs produced by other periodontal pathogens, *Tannerella forsythia* and *Treponema denticola*, induced a weak or no NOD1/2 response respectively, highlighting the different abilities of BMVs to activate NOD1 and NOD2 in the context of periodontitis (75). In contrast to pathogen derived BMVs, commensal derived BMVs produced by the commensal gut bacterium *Bacteroides fragilis*, downregulated the production of the anti-inflammatory cytokine IL-10 by NOD2 knockout murine bone marrow-derived dendritic cells (BMDCs) (76). This indicated that commensal BMVs may be involved in the regulation of anti-inflammatory immune responses in a NOD-dependent manner. Overall, in addition to PGN-containing BMVs entering host cells to initiate NOD1 or NOD2 dependent pro- or anti-inflammatory immune responses (reviewed by 77, 78), several studies have also demonstrated that bacteria can modify their PGN to subvert detection by NOD1 and NOD2, in order to increase bacterial survival and persistence in the host.

### 2.4 Subversion of NOD1 and NOD2 detection by PGN adaption

To establish an infection within the host and to limit inflammation, several bacteria have adapted mechanisms to subvert detection by NOD1 and NOD2. For example, *Listeria monocytogenes* undergoes PGN N*-*deacetylation to prevent NOD agonist presentation during intracellular infection to limit inflammation and clearance from the host (**Figure 1**) (79). Deletion of the N*-*deacetylase gene *pgdA* in *L. monocytogenes* resulted in loss of infectivity of such mutants in mice, and *L. monocytogenes pgdA* mutants were efficiently killed by murine macrophages resulting in the generation of a TLR2 and NOD1-dependent IFN-β response (79). This indicates that PGN modification by N*-*deacetylation is an effective mechanism used by *L. monocytogenes* to evade NOD detection and clearance from the host (79). Other bacterial species including *Mycobacterium tuberculosis, Mycobacterium smegmatis, Staphylococcus aureus* and *Neisseria meningitidis* have also developed mechanisms of PGN modification, including N-glycosylation, O-acetylation and amidation of muramic acid residues resulting in resistance to host lysozyme (**Figure 1**) (80–83). For example, *M. tuberculosis* reduces NOD1 activation by peptide-amidation of PGN fragments, which may be a mechanism to reduce the host inflammatory response in a NOD1-dependent manner in order to establish an effective infection in the host (83).

In addition to post-translational modifications such as O*-*acetylation which may contribute to NOD1 and NOD2 immune evasion (82), *N. meningitidis* penicillin-binding protein 2 (PBP2) is also thought to contribute to evasion of NOD1 activation (84). PBP2 is involved in PGN biosynthesis, cell elongation and increased resistance to penicillin G, and *N. meningitidis* strains with alterations to *penA* had decreased tetrapeptide-containing muropeptides, resulting in reduced NOD1 activation compared to wild-type *N. meningitidis* (**Figure 1**) (84). These strains also contained a decreased amount of the monomeric muropeptide anhydrous disaccharide-tetrapeptide, known as tracheal cytotoxin (TCT), which is known to have cytopathologic and proinflammatory properties (84) and is the key ligand of the murine NOD1 protein (26). Interestingly, *N. meningitidis* with *penA* mutations were less virulent despite their resistance to penicillin G (84). Therefore, it has been proposed that reduced TCT production, and reduced NOD1 and NOD2 activation by *N. meningitidis* strains is a disadvantage during infection, whereby cytotoxicity and inflammation are associated with the effective establishment of infection (84). Other bacteria also have inherent differences in their PGN composition which can differentially affect the activation of NOD1 and NOD2, for example the periodontal pathogen *P. gingivalis* demonstrated weaker activation of NOD1 and NOD2 compared to *Escherichia coli* and *Fusobacterium nucleatum* (85), despite *P. gingivalis* BMVs being shown to activate NOD1 and NOD2 (75). Similarly to *N. meningitidis*, it is thought that different *P. gingivalis* strains are variable in their dipeptide and tripeptide PGN content, and therefore their ability to activate NOD1 and NOD2 (85). The weak activation of NOD1 and NOD2 by *P. gingivalis* bacteria may be a mechanism to modulate host inflammatory immune responses, and therefore promote survival of pathogenic bacteria in the periodontal environment (85, 86).

*Helicobacter pylori* has also been shown to evade detection by NOD1 and NOD2, which occurs during its transition from spiral to coccoid forms (87). Spiral *H. pylori* expresses a T4SS that can inject PGN into host cells and initiate a NOD1-dependent inflammatory response but are sensitive to antibiotics and host inflammatory molecules (**Figure 1**) (30). However, coccoid forms of *H. pylori* are more resistant to antibiotics and host inflammatory assaults (reviewed by 88). The putative PGN hydrolase encoded by the *amiA* gene in *H. pylori* is thought to contribute to the accumulation of GM-dipeptide, a NOD2 agonist, during the transformation from spiral to coccoid forms (**Figure 1**) (87). Conversely, as GM-dipeptide accumulates in coccoid *H. pylori*, the NOD1 agonist GM-tripeptide is decreased (87). This suggests that switching of *H. pylori* from the spiral form to coccoid results in evasion from detection by NOD1 in human epithelial cells and escape from the host proinflammatory immune response (**Figure 1**) (87). The invasive bacterium *Legionella pneumophila* has also been shown to have mechanisms to subvert NOD1 activation (89). *L. pneumophila* infects macrophages intracellularly and has been shown to subvert NOD1 detection by expressing the protein EnhC which interferes with the bacterial protein SltL, a PGN degradative enzyme responsible for the generation of NOD1 ligand (89). By blocking the generation of NOD1 ligand, *L. pneumophila* prevents its detection by NOD1 and the generation of a proinflammatory immune response, thus contributing to bacterial viability (**Figure 1**) (89).

In addition to inherent PGN modifications that result in evasion of NOD1 and NOD2 detection, the Gram-negative pathogen *Leptospira interrogans* has been shown to express a protein that enables evasion of NOD1 and NOD2 activation (90). *L. interrogans* escapes recognition by NOD1 and NOD2 by producing a lipoprotein, LipL21, that binds to *L. interrogans* PGN and prevents the action of PGN hydrolases, resulting in sequestration of NOD agonists on the bacterial surface (**Figure 1**) (90). As NOD1 and NOD2 agonists are not released from the surface of *L. interrogans* due to the action of LipL21, *L. interrogans* is able to also escape recognition by NOD1 and NOD2 to establish an infection in the host (90). Further molecular mechanisms of NOD1 and NOD2 modulation and specific PGN biochemical modifications that affect NOD1/2 signalling are reviewed in detail elsewhere (91).

Taken together, recent advances show that NOD1 and NOD2 are much more complex than being exclusively MAMP sensors. Both NLRs can be activated by cellular stress, modulation of cellular small GTPase activity and F-actin perturbations. Bacterial pathogens have evolved multiple measures to counteract NOD activation and to adopt the inflammatory response in the host for their benefit. This includes the modification of PGN, PGN packaging by BMVs, interception of NOD1/2 signalling and targeting of small GTPases by effector proteins. It is clear that NLRs have several roles not only in the detection of bacterial PGN, but also in regulation of immunity in concert with other NLR proteins (92). In particular, bacteria can indirectly affect NLR signalling in several ways, including the inactivation of GTPases which have been shown to be important for both NOD1/2 and pyrin inflammasome signalling (92, 93). In this way, bacteria may modify their PGN in order to alter their activation of several NLRs to ultimately activate or subvert host immunity.

## 3 Inflammasome forming NLRs

The formation of multiprotein signalling complexes termed inflammasomes that consists of an NLR protein, the adaptor protein apoptosis-associated speck-like protein containing a CARD (ASC) and caspase-1, was first described by the group of Jürg Tschopp for NLRP1 (94). Inflammasome oligomerisation induces the production of active caspase-1, triggering the processing of pro-IL-1β, pro-IL-18 (95, 96) and gasdermin D, leading to pore formation, release of IL-1β and IL-18 and eventually pyroptosis (97–100).

Inflammasome formation of NLRP1, NLRP3 and NLRC4 (101–103) as well as for the non-NLR proteins AIM2 (104–106) and Pyrin (107) has been well characterised. The formation of inflammasomes was further reported for NLRP6, NLRP7, NLRP12 and NLRC5 (108–111). Recruitment of ASC by NLRP proteins is mediated through homotypic PYD-PYD interactions. ASC then recruits pro-Caspase-1 *via* homotypic CARD-CARD interactions. In this section we will focus on two of the best described inflammasome-forming NLRs: NLRP3 and NLRC4 and describe how different bacterial pathogens evade their activation.

### 3.1 The NLRC4/NAIP inflammasome

**Figure 2:**
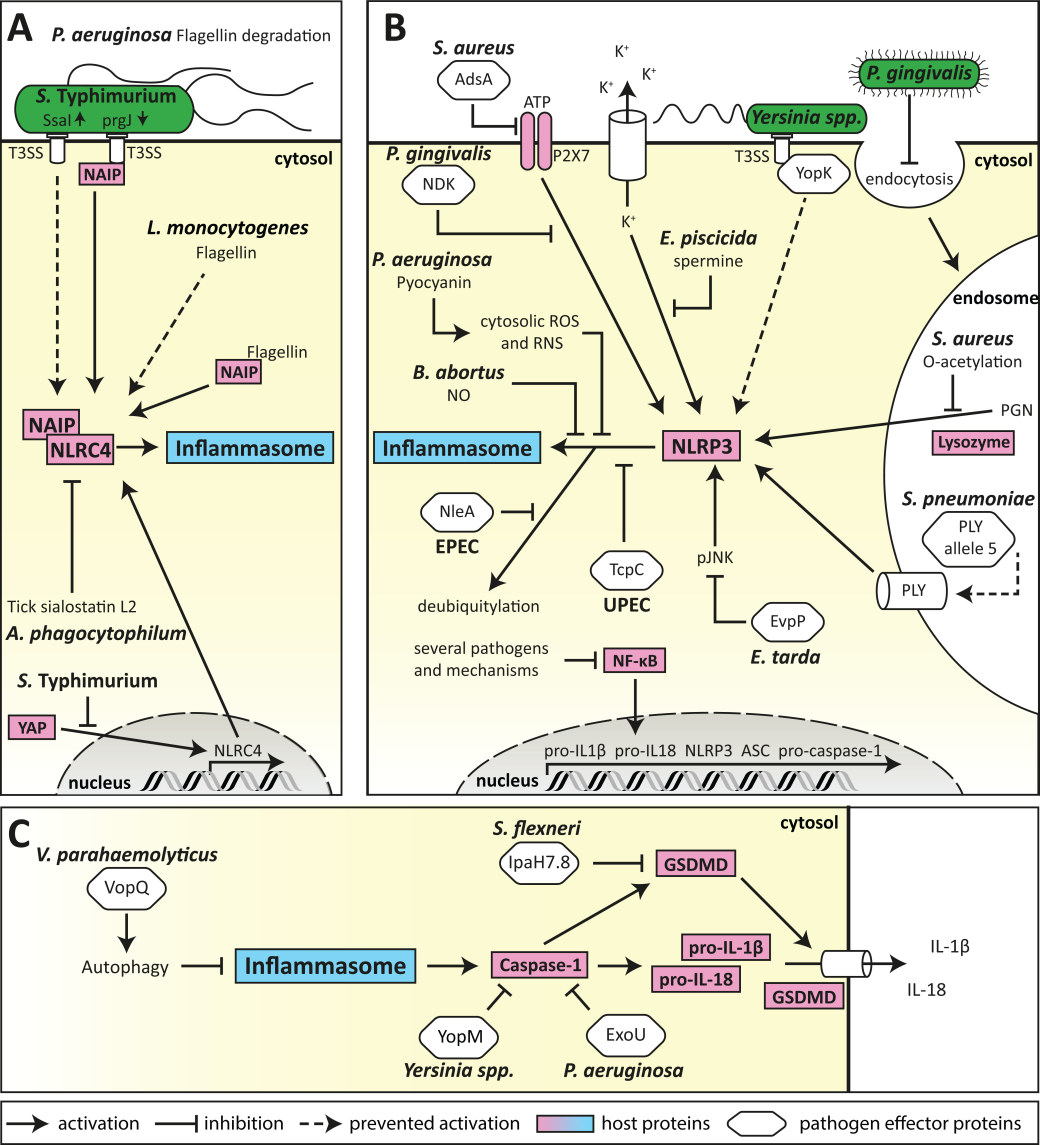
Mechanism of bacterial evasion of inflammasome activation and signalling. **(A)** Bacterial evasion of the NLRC4 inflammasome. Evasion of NAIP detection is one of the major subversion strategies for bacteria recognised by the NLRC4 inflammasome. This can be performed by the expression of poorly immunogenic *S. *Typhimurium T3SS rod proteins, or *L. monocytogenes* flagellin, as well as by proteasomal degradation of *P. aeruginosa* flagellin. Furthermore, expression of NLRC4 can be suppressed by *S. *Typhimurium through inhibition of host transcription factors, and by *A. phagocytophilum* by exploitation of vector-mediated release of anti-inflammatory compounds. **(B)** Bacterial evasion of the NLRP3 inflammasome. Subversion of the NLRP3 inflammasome can be conferred by several different mechanisms (shown in clockwise order). First, several pathogens can prevent transcription of inflammasome components by inhibiting NF-κB signalling. Second, pathogenic bacteria can inhibit activation of the NLRP3 inflammasome by DAMPs, such as *via* the degradation of extracellular ATP by AdsA from *S. aureus*, inhibition of the ATP-receptor P2X7 signalling by *P. gingivalis* NDK, or accumulation of cytosolic spermine by *E. piscicida*. Third, pathogens can evade recognition by preventing the detection of their ligands such as masking of *Yersinia* spp. T3SS effector YopK, suppression of endocytosis by *P. gingivalis*, modification of *S. aureus* PGN by O-acetylation, or expression of mutant virulence factors that lack NLRP3-activating properties, such as *S. pneumoniae* PLY. Finally, NLRP3 inflammasome formation can be targeted directly by bacterial effector proteins such as *E. tarda* EvpP or UPEC TcpC, and by EPEC NleA-mediated deubiquitylation as well as by *P. aeruginosa* pyocyanin or *B. abortus*-derived nitric oxide (NO). **(C)** Targeting mechanisms common to NLRP3 and NLRC4 inflammasome formation allow pathogens to efficiently prevent secretion of IL-1β and IL-18. ASC-speck formation can be prevented by induction of autophagy by *V. parahaemolyticus* VopQ. Caspase-1 can be directly targeted by bacterial effector proteins such as *Yersinia* spp. YopM, or *P. aeruginosa* ExoU, to prevent proteolytic processing of pro-IL-1β, pro-IL-18 and gasdermin D (GSDMD). GSDMD is further targeted by *S. flexneri* IpaH7.8 for degradation, preventing NLRC4-mediated pore-formation.

A unique NLR-NLR interaction exists between the intracellular receptor neuronal apoptosis inhibitory proteins (NAIP) and inflammasome adaptor protein NOD-LRR-and CARD-containing 4 (NLRC4) that form the NLRC4/NAIP inflammasome (112, 113). The NAIP thereby serve as sensors to detect specific bacterial-derived MAMPs, namely the inner rod proteins of the bacterial type III secretion system (T3SS), and flagellin [reviewed in (102)]. NAIP/NLRC4 activation occurs in response to the delivery of their specific ligands *via* the bacterial T3SS or T4SS (114), flagella-containing bacterial membrane vesicles (115), or the presence of intracellular pathogens (116). NAIP receptors were first observed as being critical in the defence against infection by the intracellular pathogen *L. pneumophila*, whereby it was observed that murine macrophages harbouring a mutation in the Lgn1 locus, which encodes the *Naip5* gene, were susceptible to *L. pneumophila* infection (117–119). Furthermore, expression of NLRC4 has been shown to be critical in defence against enteric pathogens including *S.* Typhimurium (120), *E. coli* (121) and *S. flexneri* (122), as well as systemic pathogens such as *L. pneumophila* (123), *Pseudomonas aeruginosa* and *K. pneumoniae* (124, 125). Mice express four NAIP receptors, namely NAIP1 and NAIP2 that detect T3SS inner rod proteins, and NAIP5 and NAIP6 that detect flagellin; while humans express a single NAIP with splice variants that detect both T3SS proteins and flagellin [reviewed in (126)]. The NLRC4 inflammasome is especially important during infection of intestinal epithelial cells (127), and its expression can be upregulated by pro-inflammatory stimuli, such as TNFα (128). Following an initial priming signal generally involving the activation of TLRs, the ligand-triggered activation of NAIP initiates co-oligomerization with the NLRC4 adaptor to form a multiprotein inflammasome complex, culminating in a potent inflammasome response hallmarked by production of active caspase-1, IL-1β and IL-18, as well as pyroptosis [reviewed in (102)]. NLRC4 is different to other NLRPs as it can recruit caspase-1 independently of ASC through CARD-CARD interaction, however ASC is nucleated by NLRC4 and can greatly enhance caspase-1 activation (15). Pathogenic bacteria have co-evolved counter mechanisms to either avoid detection by NAIP, prevent NLRC4 signalling, exploit the NLRC4 pathway to the benefit of the pathogen, or dampen the inflammasome response (**Figures 2A**, **C**). In addition, dampening of NAIP-NLRC4 activation is thought to be critical for promoting immunotolerance to enteric commensal bacteria.

#### 3.1.1 Evasion of detection by NAIP

Several pathogens evade NAIP detection by reducing the accessibility of ligands. When intracellular, *S.* Typhimurium represses expression of the flagellin protein FliC through the expression of the protease ClpXP, allowing the pathogen to transverse the epithelial barrier undetected (129). *S*. Typhimurium also impedes clearance from macrophages by reducing expression of the immunogenic T3SS rod protein PrgJ, in favour of the poorly immunogenic SsaI rod proteins (**Figure 2A**) (130). In addition, *L. monocytogenes* evades detection by expressing flagellin that is a poor activator of NLRC4 (**Figure 2A**) (131), while *P. aeruginosa* secretes proteases that degrade extracellular flagellin (**Figure 2A**) to limit TLR5 activation, however whether this mechanism also leads to evasion of NLRC4 inflammasome sensing is unknown. These evasion mechanisms enable pathogens to remain undetected by the NAIP, and thereby facilitate host colonisation.

#### 3.1.2 Blocking NLRC4 signalling

Several pathogens block NLRC4 signalling to prevent inflammasome-mediated cytokine production or pyroptosis. *S.* Typhimurium can directly modulate the host response by downregulating NLRC4 expression in infected B-cells (132). This is mediated by phosphorylation of the host transcriptional activator Yap, thereby preventing its nuclear translocation and transcriptional activation of NLRC4 (**Figure 2A**) in a process depended on the *Salmonella* pathogenicity island 1 (SPI1) T3SS (132). Furthermore, a unique subversion mechanism is utilised by the tick-borne pathogen *Anaplasma phagocytophilum* which profits from the anti-inflammatory tick salivary protein sialostatin L2. Sialostatin L2 blocks NLRC4 oligomerisation and prevents caspase-1 activation, thereby preventing an inflammasome response to *A. phagocytophilum* (**Figure 2A**) (133). This represents a unique cross-kingdom mechanism that allows the bacterial pathogen to establish colonisation of the human host (133). Collectively, these studies reveal the sophisticated mechanisms employed by pathogens to block NLRC4 signalling at different points in the inflammasome pathway.

#### 3.1.3 Exploitation of NLRC4 activation

An alternative method of subverting the host response is to exploit it for the benefit of the pathogen. The gastric pathogen *H. pylori* induces NLRC4 activation in gastric epithelial cells mediated by its type IV secretion system, which results in the inhibition of the Th17/IL-17 response and downregulation of beta defensin-1 (BD-1), leading to reduced killing of *H. pylori* (134). NLRC4-deficient mice were found to be more adept at clearing *H. pylori* infection, highlighting the importance of this subversion mechanism in *H. pylori* colonisation and persistence (134). Similarly, activation of NLRC4 by *S. aureus* in murine lung epithelial cells was shown to impair IL-17A-dependent neutrophil recruitment (135), preventing bacterial clearance from the lungs. In contrast, NLRC4-deficient mice displayed increased bacterial clearance and improved host survival, highlighting the vital role this subversion mechanism plays in *S. aureus* colonisation (135).

During *S.* Typhimurium infection in mice, NLRC4 is activated by flagellin of the bacteria (136). An elegant study showed that NLRC4 activation can affect adaptive immunity by reducing CD4^+^ T-cell-mediated immune memory (136). In NLRC4-deficient animals as well as in animals infected with an *S.* Typhimurium strain that expressed a form of flagellin that cannot activate NLRC4, higher levels of IFN-γ secreting Th1 cells and memory CD4^+^ T-cells were observed (136). The exact mechanism remains elusive but involves activation of NLRP3 (136).

These studies illustrate that PRR activation can have consequences beyond the direct innate response that can be detrimental to the host. Understanding of these complex interactions between the innate and adaptive immune system will be essential to gaining insight into their role in immunopathology and infectious disease towards specific pathogens.

#### 3.1.4 Dampening of the NLRC4 response to facilitate immunotolerance

Dampening of the NLRC4 response has also been linked to facilitating immunotolerance to commensal bacteria (137). Studies have shown that the uptake of free flagellin by intestinal phagocytes leads to an adaptive immune response that inhibits the NLRC4 response, which is thought to promote immunotolerance to commensals. Similarly, a study showed that uptake of commensal bacteria by intestinal phagocytes did not lead to activation of NLRC4, yet uptake of the pathogens *S. *Typhimurium or *P. aeruginosa* triggered NLRC4-mediated production of mature IL-1β, suggesting the NAIP-NLRC4 system can discriminate between pathogenic and non-pathogenic bacteria (137). More studies are required to determine the mechanisms involved in regulating NAIP-NLRC4 activation and signalling that tailors the host response to commensals or pathogens and the bacterial factors involved.

### 3.2 The NLRP3 inflammasome

Canonical formation of the NLRP3 inflammasome requires two distinct signals. First, a priming signal leads to NF-κB-induced transcription of the inflammasome components, as well as pro-IL-1β and pro-IL-18. A second activation step then induces the formation of the inflammasome and activation of caspase-1. The second signal can be conferred by a broad range of stimuli which induce extensive changes in cellular homeostasis. These stimuli include extracellular ATP, lysosomal rupture by crystalline structures, mitochondrial ROS, pore formation and changes in the K^+^ or Ca^2+^ homeostasis, (reviewed in (101)). Interestingly, flagellin can also activate the NLRP3 inflammasome indirectly in a ROS- and cathepsin-dependent manner (138), suggesting that ROS is a central activator linking NLRP3 to bacterial detection.

NLRP3 is found predominantly in myeloid cells and its activation is a tightly regulated mechanism (reviewed in (101)). Excessive inflammasome activity is associated with systemic auto-inflammatory syndromes, termed cryopyrin-associated periodic-syndromes (CAPS) (139). Regulation of NLRP3 is orchestrated by several post translational modifications including deubiquitylation, selective phosphorylation and dephosphorylation as well as degradation of small ubiquitin-related modifier (SUMO) known as deSUMOylation (101). Furthermore, interaction partners that are critical for NLRP3 inflammasome activation have been identified, such as the kinase NEK7 (140, 141) and the RNA-helicase DDX3X (142).

The NLRP3 inflammasome can additionally be activated by non-canonical mechanisms involving caspase-11 in mice and caspase-4/5 in humans (143–146). Direct sensing of LPS by those caspases (147, 148) results in caspase activation and subsequent cleavage of gasdermin D, releasing its N-terminal fragment which forms pores in the cell membrane and induces a form of lytic cell death, termed pyroptosis (98). The cleaved p30 gasdermin D fragment then leads to cell-intrinsic activation of NLRP3 (143).

Gasdermin D is also cleaved upon caspase-1 activation by classical NLRP3 activation to allow for the release of IL-1β and IL-18. This also leads to induction of pyroptosis, a highly pro-inflammatory form of cell death, as the cellular contents of pyroptotic are released and can act as DAMPs. Additionally, IL-1β and IL-18 are among the most potent pro-inflammatory cytokines with multiple functions, including the induction of fever, and available data suggests that in most cells the NLRP3 inflammasome is the main platform for caspase-1 activation. It is hence not surprising that several pathogens have evolved subversion mechanisms to evade NLRP3-induced immune responses. As bacterial subversion mechanisms of NLR- and TLR-induced NF-κB signalling have been extensively reviewed in the literature (49, 50), we will focus on strategies directly targeting the activation and function of the NLRP3 inflammasome (**Figures 2B**, **C**).

### 3.3 Evasion of NLRP3-mediated recognition

Although NLRP3 is activated by a broad range of DAMPs, several bacterial pathogens have evolved mechanisms to evade detection by reducing the generation of NLRP3 activating stimuli. Phagocytic internalisation and lysozymal degradation of particulate *S. aureus* PGN is known to induce NLRP3-dependent IL-1β secretion from murine bone marrow-derived macrophages (BMDMs) independently of NOD2, yet the cell wall of pathogenic *S. aureus* strains has been shown to be highly resistant to lysozyme (149), due to O-acetylation of PGN. This modification prevents NLRP3 activation, IL-1β secretion and ultimately reduces macrophage-mediated killing of *S. aureus* (81) (**Figure 2B**). Additionally, *S. aureus* surface enzyme adenosine synthase A (AdsA) degrades ATP, ADP, and AMP to adenosine, thereby preventing NLRP3 activation by extracellular ATP (150) (**Figure 2B**). These subversion strategies allow *S. aureus* to remain undetected by the NLRP3 inflammasome, facilitating colonisation of the host and preventing bacterial killing.

Similarly, the emerging *S. pneumoniae* serotype 1 MLST306 and serotype 8 MLST53 strains have been described to evade NLRP3 inflammasome detection (151) by expression of an altered version of the endotoxin pneumolysin (PLY) (152). While retaining other virulence-related functions (153–157), this PLY lacks pore-forming ability (158) which strongly reduces IL-1β induction, thereby reducing bacterial killing (159) (**Figure 2B**). Furthermore, while the *Y. pseudotuberculosis* T3SS effectors YopB and YopD induce NLRP3 inflammasome activation by a poorly understood mechanism, translocation of these bacterial proteins is tightly controlled by YopK during infection, which inhibits exessive translocation of these effectors (160, 161) and therefore limits NLRP3 activation (162) (**Figure 2B**). This exemplifies the co-evolution of pathogen and host, resulting in elegant mechanisms of the pathogen to fine-tune inflammasome regulation for the benefit of host fitness and bacterial replication.

### 3.4 Metabolic interference with NLRP3 inflammasome formation

Recently, the role of several metabolites and secondary messenger molecules in modulation of innate immune receptors has been identified. For example, nitric oxide (NO) (163) and the Krebs cycle derived metabolite itaconate (164) have been described as inhibitors of the NLRP3 inflammasome. It is hence not surprising that several bacterial pathogens alter cellular metabolism for their benefit.

Nitrate reduction to di-nitrogen by *Brucella abortus* has been demonstrated to result in the presence of intermediate NO in iNOS-deficient cells and thus inhibition of the NLRP3 inflammasome (165) (**Figure 2B**). Furthermore, upon macrophage engulfment, the fish pathogen *Edwardsiella piscicida* delivers spermine to the cytosol in a T3SS-dependent manner, mediated by recruitment of arginine importer cationic acid transporter 1 (mCAT-1) and putrescin exporter organic cation transporter 2 (Oct-2) to the bacteria-containing vacuole (166). Cytosolic accumulation of spermine then inhibits the K^+^ efflux-dependent activation of the NLRP3 inflammasome (166, 167) (**Figure 2B**). These studies demonstrate how the interplay between bacterial and host metabolism can regulate innate immune responses.

### 3.5 Direct targeting of NLRP3 by bacterial effector proteins

Suppression of NLRP3 activation is a common subversion strategy among several pathogens. This occurs by either the interference with the second signal of NLRP3 inflammasome activation, or by direct targeting of NLRP3 itself. Inhibition of the second signal is often conferred by preventing alterations of cellular homeostasis that are necessary for NLRP3 activation. This is seen for example in *P. gingivalis* infection where secreted nucleoside diphosphate kinase homologue (NDK) supresses NLRP3 inflammasome formation upon recognition of ATP through the P2X purinoceptor 7 (P2X7). Here, NDK seems to establish an anti-oxidative environment, limiting ATP-induced mitochondrial ROS production (168) (**Figure 2B**). Similarly, the *Edwardsiella tarda* T6SS effector protein EvpP inhibits activation of the NLRP3 inflammasome by counteracting the cytoplasmic Ca^2+^ increase, necessary for c-Jun NH2-terminal protein kinase (Jnk) activation, however the exact mechanism by which EvpP confers its effect is still unclear (169) (**Figure 2B**).

Direct interaction with NLRP3, or alteration of its post transcriptional modification (PTM) have also been described as subversion mechanisms for several pathogens. The enteropathogenic *E. coli* (EPEC) effector protein NleA interacts with NLRP3 and prevents its de-ubiquitination, resulting in reduced caspase-1 recruitment to the NLRP3 foci (170) (**Figure 2B**). Similarly, direct interaction of Toll/IL-1 receptor containing protein C (TcpC) from uropathogenic *E. coli* (UPEC) with NLRP3 and caspase-1 in BMDMs inhibits NLRP3-inflammasome induced IL-1β secretion (171) (**Figure 2B**). Furthermore, the pigment phenazine pyocyanin (PCN) produced by *P. aeruginosa* acts as a virulence factor that generates superoxide by the transfer of electrons from NADH and NADPH to oxygen. It was shown that PCN-derived ROS and RNS can lead to specific inhibition of the NLRP3 inflammasome by post-translationally blocking both ASC speck formation in BMDMs (172) and subsequent IL-1β secretion (169). In this manner, *P. aeruginosa* evades immune recognition and escapes macrophage-mediated killing (172). These studies highlight the broad yet highly effective range of effector functions through which bacterial pathogens prevent NLRP3 inflammasome formation.

Inhibition of the NLRP3 response is beneficial for bacterial fitness, as mutant strains lacking NLRP3 subversion mechanisms, in general show reduced survival *in vivo* (149, 173, 174). However, while activation of the NLRP3 inflammasome benefits the host by facilitating bacterial clearance, it can also lead to detrimental effects for the host. It has been shown that the increased clearance of *S. aureus* mutants, incapable of NLRP3 subversion, can lead to the appearance of skin lesions at the site of subcutaneous infection, indicating enhanced host-response-mediated tissue damage (149). Furthermore, activation of the NLRP3-inflammasome has been shown to drive immunopathology in *Bacillus cereus* infection, where NLRP3-induced inflammation strongly enhances the mortality of infected mice (175) and in pneumococcal meningitis, driven by IL-18 and IFN-γ (176, 177). Thus, while NLRP3-suppression generally is beneficial for pathogen survival, it can also be beneficial to limit tissue damage in the host. Overall, the importance of the NLRP3-inflammasome in the antibacterial immune response is highlighted by the broad range of pathogens which subvert its activation and effects for better survival in the host. However, although the general mechanisms of inflammasome activation appear to be highly conserved between mice and humans, there are differences in the relative importance of singular components of the multifaceted immune response (178). Overall, the translation from findings regarding NLR activation in mouse models into the human setting must be evaluated critically.

### 3.6 Subversion of inflammasome effector mechanisms

In the response against pathogens, co-operation of several inflammasomes will happen in the host and is often necessary to facilitate bacterial clearance (179). Yet to counter the host’s multi-faceted response, many pathogens have evolved subversion strategies to target general mechanisms that prevent the assembly, activation, or signalling of several inflammasomes.

Suppression of inflammasome assembly is utilised by several pathogens. The *P. aeruginosa* quorum sensing-regulated virulence factor PCN and autoinducer 3-oxo-C12-homoserine lactone suppress the assembly and activation of both the NLRP3 and NLRC4 inflammasomes (180). Similarly, *S. *Typhimurium can suppress the activation of the NLRP3 and NLRC4 inflammasomes in human macrophages by a hitherto unknown SPI2 T3SS secreted effector to prevent IL-1β production and cell death, allowing bacterial persistence in macrophages (181).

Inhibition of the inflammatory caspases is another central mechanism employed by several bacterial species for immune evasion. For example, *Yersinia pestis* expresses a broad range of effector proteins that can target caspase-1 activation through different mechanisms, such as sequestration and inhibition of auto-proteolytic processing by YopM (182) or through inactivation of Rho GTPases by YopE and YopT (183, 184) (**Figure 2C**). *P. aeruginosa* secretes a phospholipase enzyme exoenzyme U (ExoU) that inhibits caspase-1 activity to block NLRP3 and NLRC4 inflammasome signalling (124) (**Figure 2C**). *S. flexneri* for example can block the non-canonical inflammasome by posttranslational modification of caspase-4 by its T3SS effector OspC3 using the uncommon ADP riboxanation to prevent cell death and inflammatory cytokine production upon intracellular LPS sensing (185, 186).

Pathogens can also block cell death to allow them to persist in host cells. For example, *S. flexneri* secretes the ubiquitin ligase IpaH7.8 *via* its T3SS, which cleaves gasdermin D to prevent NLRC4-mediated pyroptosis (**Figure 2C**). This allows the bacteria to persist in human epithelial cells, while also preventing the release of danger signals to limit the activation and recruitment of immune molecules (187). While IpaH7.8 has only been shown to block NLRC4-mediated pyroptosis, it remains to be seen whether it can block broader activation of pyroptosis.

To reduce inflammasome signalling, pathogens can also exploit the host cellular degradation process of autophagy to degrade effector molecules released upon inflammasome activation, a mechanism recently termed “inflammophagy” that is also used by the host cell to control innate immune responses (188). The *Vibrio parahaemolyticus* T3SS effector protein VopQ induces autophagy in infected macrophages, which interferes with ASC speck formation to suppress NLRC4 and partially suppress NLRP3 signalling (189) (**Figure 2C**). Furthermore, the phosphothreonin lyase SpvC of *S.* Typhimurium was suggested to dampen xenophagy and induce autophagy-dependent degradation of NLRP3 and NLRC4, albeit the exact mechanism remains elusive (190).

Furthermore, a given pathogen will likely activate multiple PRRs in the host, and therefore, to facilitate host colonisation, pathogens have evolved mechanisms to subvert a broad range of PRRs in addition to inflammasomes. For example, infection of macrophages with *B. abortus* that are deficient in NO production, which is known to inhibit NLRP3, resulted in higher secretion of IL-1β, but no differences in bacterial load were observed, indicating that *B. abortus* employs additional mechanisms to ensure survival in macrophages (165). Similarly, although recognition of *Y. pestis* T3SS by the NLRP3 inflammasome was important for the caspase-1 response observed in cultured BMDMs, bacterial colonisation levels of *Y. pestis* were unaltered between WT and *Nlrp3* ^-/-^ mice (162). These studies suggest that although inflammasome activation is central to the response against several pathogens, a multifaceted response is required to successfully prevent host colonisation. Taken together, pathogens have evolved multiple mechanisms to avoid inflammasome detection and signalling, to facilitate colonisation, and to promote persistence in the host.

### 3.7 Therapeutic exploitation of inflammasome subversion

It is interesting to speculate whether bacterial subversion of inflammasome activation and signalling could be harnessed for the alleviation of inflammasome-driven diseases. *Lactobacillus paracasei*, a strain of the lactic acid bacteria commonly used as a probiotic, has been shown to dampen the activation of the NLRP3, as well as the NLRC4 and AIM2 inflammasomes, by induction of IL-10 *via* NOD2 in BMDMs (191). In initial studies, oral administration of *L. paracasei* strain KW3110 has been used *in vivo* to reduce NLRP3-dependent neutrophil recruitment in monosodium urate (MSU)-induced peritonitis of C56BL/6 mice and improve insulin sensitivity in high fat diet (HFD) fed, obese mice (191). Additionally, oral administration of KW3110 reduced T-cell infiltration of visceral adipose tissue in HFD fed mice (191), an NLRP3-dependent mechanism which contributes to insulin resistance (192). General evasion of inflammasome activation by *P. gingivalis* through suppression of endocytosis can also prevent inflammasome activation by *E. coli, F. nucleatum*, or DAMPs and PAMPs delivered by endocytosis (193), further indicating a potentially complex regulatory network which has developed within the microbiota that may be harnessed for therapeutic applications.

## 4 Conclusion

NLR proteins are host sensors for bacterial pathogens and recent advances have shown that NOD1/2, NLRC4/NAIP and NLRP3 are physiological relevant PRRs in mammals. Bacterial pathogens co-evolved with these proteins in order to establish a fruitful balance of the immune response to support both fitness of the host and replication of the pathogen. Subversion strategies used by bacteria to avoid NLR activation include the use of modification and reduced release of their PAMPs, targeting of the receptors and their pathway components as well as sophisticated use of the immune response of the host to dampen adaptive immune functions. Here we discussed the most prominent examples of bacterial subversion of the key NLR protein pathways. Albeit most studies concentrated on individual NLRs or bacterial components and effector proteins, bacteria can activate a multitude of PRRs, produce several MAMPs, and can release a large range of effector proteins that can result in a much more complex scenario of immune activation and inhibition in the host. Therefore, future studies using novel holistic technological approaches to delineate the molecular details of host-pathogen interactions both in complex models and at the single cell level will allow us to gain insights regarding systemic and adaptive immune responses and metabolic alternations related to the activation of host PRRs by bacterial pathogens. Ultimately, this will be helpful for defining new therapeutic strategies and treatments for infectious disease and their prevention by vaccination.

## Author contributions

TAK and MK-L conceived and edited the manuscript and assured funding. IK wrote the first draft, edited the manuscript and generated the figures. EJ and NB wrote the first draft and edited the manuscript. All authors contributed to the article and approved the submitted version.

## Funding

MK-L is funded by the Australian Research Council (Discovery Project DP190101655) and a veski Inspiring Women Fellowship. This work was supported by a mobility grant from the German Academic Exchange Service (DAAD), grant PPP 57445802 to TAK and MK-L.

## Acknowledgments

We apologize to all colleagues whose excellent work could not be included due to space limitations.

## Conflict of interest

The authors declare that the research was conducted in the absence of any commercial or financial relationships that could be construed as a potential conflict of interest.

## Publisher’s note

All claims expressed in this article are solely those of the authors and do not necessarily represent those of their affiliated organizations, or those of the publisher, the editors and the reviewers. Any product that may be evaluated in this article, or claim that may be made by its manufacturer, is not guaranteed or endorsed by the publisher.
